# Micro-habitat distribution drives patch quality for sub-tropical rocky plateau amphibians in the northern Western Ghats, India

**DOI:** 10.1371/journal.pone.0194810

**Published:** 2018-03-26

**Authors:** Christopher J. Thorpe, Todd R. Lewis, Siddharth Kulkarni, Aparna Watve, Nikhil Gaitonde, David Pryce, Lewis Davies, David T. Bilton, Mairi E. Knight

**Affiliations:** 1 Ecology and Evolution Research Group, School of Biological and Marine Sciences, University of Plymouth, Drake Circus, Plymouth, Devon, United Kingdom; 2 Westfield, Wareham, Dorset, United Kingdom; 3 George Washington University, Washington D.C., United States of America; 4 Tata Institute of Social Sciences, Taljapur, Osmanabad, Maharashtra, India; 5 National Centre for Biological Sciences, Rajiv Gandhi Nagar, Kodigehalli, Bengaluru, Karnataka, India; University of Waikato, NEW ZEALAND

## Abstract

The importance of patch quality for amphibians is frequently overlooked in distribution models. Here we demonstrate that it is highly important for the persistence of endemic and endangered amphibians found in the threatened and fragile ecosystems that are the rocky plateaus in Western Maharashtra, India. These plateaus are ferricretes of laterite and characterise the northern section of the Western Ghats/Sri Lanka Biodiversity Hotspot, the eighth most important global hotspot and one of the three most threatened by population growth. We present statistically supported habitat associations for endangered and data-deficient Indian amphibians, demonstrating significant relationships between individual species and their microhabitats. Data were collected during early monsoon across two seasons. Twenty-one amphibian taxa were identified from 14 lateritic plateaus between 67 and 1179m above sea level. Twelve of the study taxa had significant associations with microhabitats using a stepwise analysis of the AICc subroutine (distLM, Primer-e, v7). Generalist taxa were associated with increased numbers of microhabitat types. Non-significant associations are reported for the remaining 9 taxa. Microhabitat distribution was spatially structured and driven by climate and human activity. Woody plants were associated with 44% of high-elevation taxa. Of the 8 low-elevation taxa 63% related to water bodies and 60% of those were associated with pools. Rock size and abundance were important for 33% of high elevation specialists. Three of the 4 caecilians were associated with rocks in addition to soil and stream presence. We conclude the plateaus are individualistic patches whose habitat quality is defined by their microhabitats within climatic zones.

## Introduction

**Fig 1:**
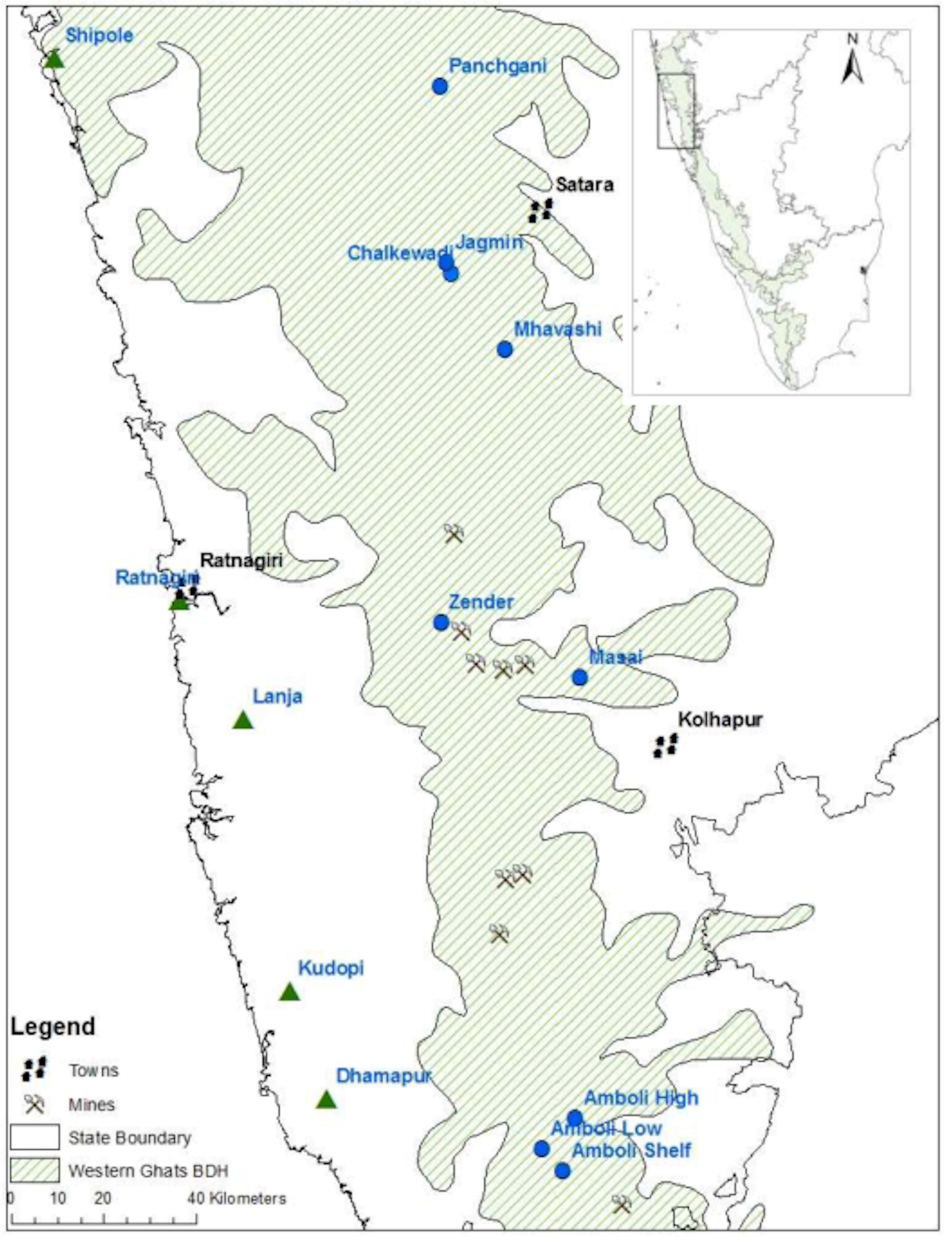
Map of study sites within the study area inset with location within India. Green triangles denote surveyed site locations below the Western Ghats escarpment and blue circles sites above it. Some mine site locations are included to illustrate the proximity of threat of mining. The biodiversity hotspot outline is derived data downloaded from ArcGIS, Environmental Systems Research Institute, Redlands, California, USA.

**Fig 2:**
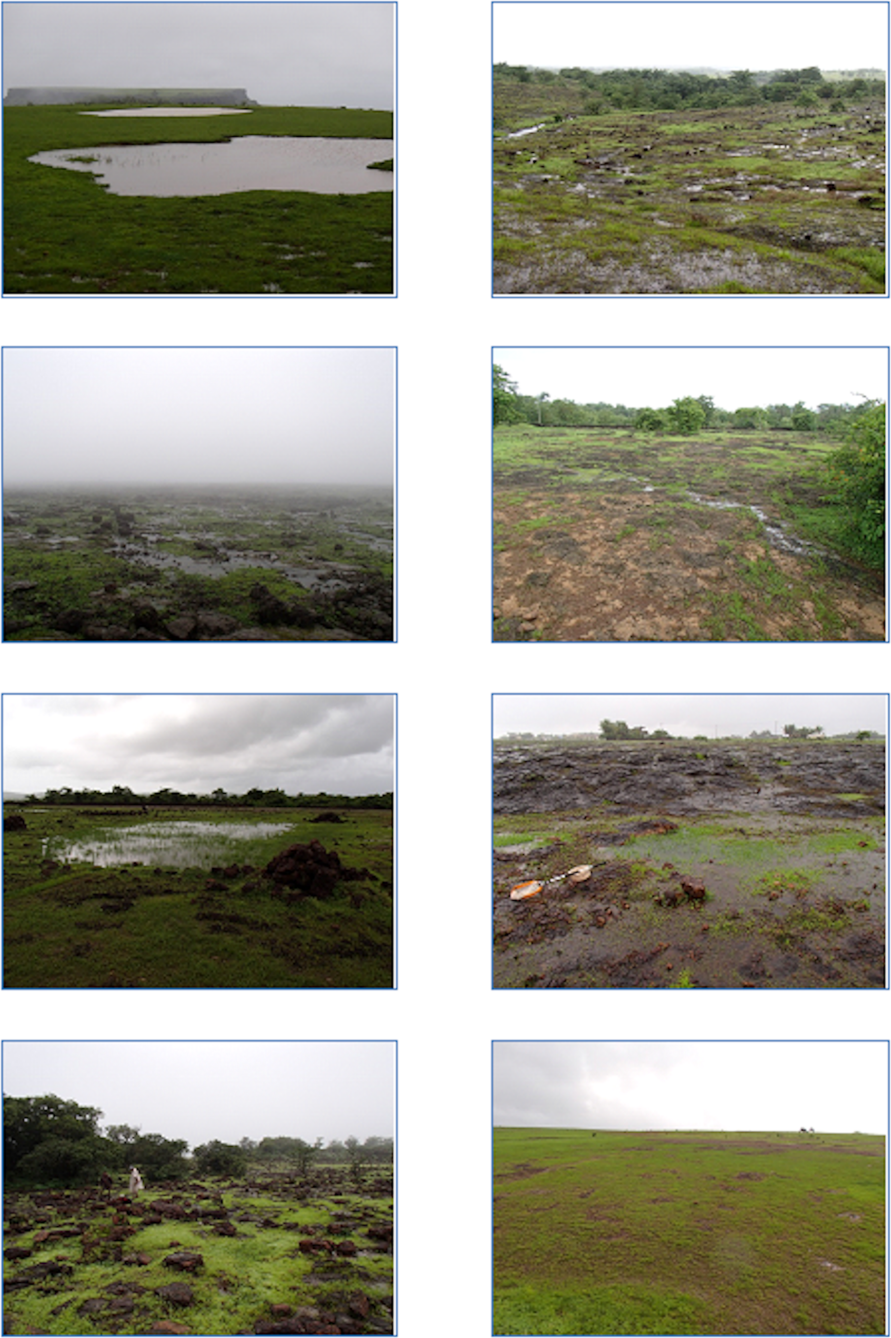
Illustrations of the varied microhabitats present on the lateritic plateaus of western Maharashtra, NWG.

The Western Ghats-Sri Lanka Biodiversity hotspot is the eighth hottest global biodiversity hotspot and one of the three most threatened by human population growth [1–3]. The northern section of the Western Ghats (NWG) is unique, being geologically distinct and biologically isolated from the central and southern sections of the Western Ghats (WG) on the Indian peninsular (Fig 1; [4–7]. Its’ rich amphibian fauna contains many critically endangered, endangered species and data deficient species [8, 9]. The area is characterised by rocky flat mesa-like hilltop ‘plateaus’ formed from ferricretes of laterite, a rock like material with a high metal content (Fig 2; [10–12]). The individual ‘plateau’ habitat is a complex matrix of microhabitats. The availability of each microhabitat varies between plateaus, but some macroscale patterns are evident. The plateaus are set within a landscape of varying complexity [13, 14]. Rocky plateaus are of international importance for their substantial contribution to regional biodiversity and endemism [15–17] and are globally threatened ecosystems [15, 18]. Those in the NWG are recognised as threatened and vulnerable ecosystems [19].

Plateau biodiversity is under pressure from demands placed by on its habitats by the rapidly growing human population. Proximate threats, that impact habitat availability, falling into two broad categories: localised threats such as illegal hunting, extraction of non-timber forest products (NTFPs), livestock grazing, and forest fires, and landscape-level threats such as mining, road construction, hydro power projects, wind farms, large-scale agricultural intensification including the creation of monoculture plantations and tourism [3, 20, 21]. Open-cast bauxite mining is a significant current threat in the NWG [22, 23]. Current mine restoration policy does not identify the importance of preserving the pre-existing habitat or its mix of microhabitats [13].

The WG is home to 161 of India’s 419 amphibians in only 5% of its land area, making the WG the most amphibian rich land area in India [24–26]. In the WG they are a highly diverse group displaying exceptional levels of endemism (87%; [3, 24, 27]. Many are endemic with a very localised distribution resulting from their Gondwanan vicariant origin, having speciated *in situ* because of topographical isolation and diverse ecological pressures [24, 28–31]. The resolution of their taxonomy is improving but in common with much of the paleo tropics little has been published on their ecology including their habitat requirements and the environmental factors regulating their distribution (but see: [25, 32–34].

Amphibian distribution is known to be highly dependent upon habitat features (see e.g. [21, 35]) resulting in their populations being spatially determined by microhabitat availability. In tropical regions the diversity of specialist micro-environments facilitates elevated levels of species richness and endemism through heterogeneity in: seasonality or microclimate [36], gradients in precipitation [37, 38], soil moisture [39, 40] and elevation [41, 42]. Evidence for this in the WG comes from the high number of reproductive strategies with at least 40 different reproductive strategies currently recognised in the region [43–48], each using different habitat resources for mate advertising, mating, egg deposition, spawning, tadpole and neonate feeding. In addition to their dependence on specific microhabitats the amphibians of the WG are threatened by the fungal pathogen *Batrachochytrium dendrobatidis* [49]. To mitigate these risks, networks of suitable sites with adequate connectivity are necessary [50].

There is a globally recognised shortfall in amphibian population and ecological data [51]. To address this the Amphibian Conservation Action Plan (ACAP) was devised by the International Union for the Conservation of Nature (IUCN) in 2005 to prioritise research directions [51]. Two of the four key strategies of ACAP are to understand the causes of population declines and changes in diversity [51]. To achieve this, baseline data are required on their ecological requirements [52, 53]. Specifically, it is highlighted as essential to identify the key environmental and habitat resources required by each taxon [54–56]. The IUCN recognise the importance of preserving habitat to conserve species [57]. However, the IUCN do not adequately account for habitat specificity in heterogeneous topography, resulting in the ranges of many species being overstated [58]. At present it is almost impossible to assess the conservation status of the amphibians of the WG due to a serious deficiency in ecological data. It is likely WG amphibians reflect the global pattern where the group is declining because of one or more stressors which frequently work synergistically: climate change; habitat destruction; pollution; over-harvesting; alien species introduction and/or disease [59–61]. Further work is urgently needed in the WG in the face of climate change scenarios and alterations in land use [62].

Amphibian patch occupancy is dependent upon patch quality and regional factors including patch distribution, the nature of the intervening landscape, and climate [63]. The importance of patch quality in regulating species distribution is becoming widely recognised as a key factor [64]. Many species distribution studies only consider landscape scale processes as climate suitability when even with a suitable climate species may be excluded by smaller scale processes including microhabitat availability [64, 65]. We propose the availability of a suitable suite of microhabitats on a plateau define its quality and are a result of variations in macroclimate, edaphic processes and anthropogenic activities [13].

Given that the NWG are part of a key priority for conservation, and that their amphibians are part of a data deficient globally vulnerable group, it is imperative that this shortfall in ecological data is urgently addressed. That urgency is acute for the NWG rocky plateaus, as these fragile ecosystems are being rapidly lost and damaged by human activity and are home to critically endangered endemic species [24, 25, 66]. As the first quantitative study of rocky plateau amphibian habitat associations, the data herein will serve as a baseline to help in forming evidence-based conservation decisions [25, 34].

## Materials and methods

### Study area

The study investigated microhabitat associations of individual amphibian taxa on the isolated lateritic plateaus in the NWG (Fig 1). These island-like plateaus are dominated by areas of exposed rock but contain a varied mixture of other habitats forming a heterogenous mosaic (Fig 2). The study focussed on 14 representative lateritic plateaus in the areas both above and below the North-South trending escarpment in the northern section of the Western Ghats/Sri Lanka Biodiversity Hotspot in western Maharashtra. The study area extends over 2° latitude (15.89°-17.92°N) and a 1112 m change in plateau elevation (67–1179 m above sea level [m]). Above the escarpment the plateaus are raised hilltop carapaces elevated from the ecologically contrasting countryside.

As temperature, rainfall seasonality and rainfall amount varies across the survey area, for comparative purposes the area was sub-divided into 2 Regions (High and Low), separated by the escarpment. Each region was further subdivided into three arbitrary latitudinal sections: North, Central and South. These are referred to as ‘eco-zones’ (similar to life-zones but in the absence of specific environmental data for the plateaus the term eco-zone is preferred [67]. Rainfall across this area ranges from <2000 mm per annum on low sites to >6000 mm on high sites peaking at >9000 mm on one high site [12, 68, 69].

These sites encompass a range of land-uses (Fig 1; Table 1). As anthropogenic disturbance within a patch is likely to change the availability of some microhabitats its type was recorded, and an arbitrary metric calculated by summing the number of disturbance factors observed on each site (Table 1). Although the figure is arbitrary, no relevant literature exists, and it allows for initial between patch comparisons. Disturbance factors recorded were; removal of loose rocks, surfaced road, unsurfaced road, built structures on the plateau, domesticated animal grazing, surfaced road within 200m of plateau, tourism, part conversion to plantation, adjacent built structures, importation of topsoil. Sites with 0–3 factors were considered to have low levels of disturbance, 4–7 Medium Disturbance, 8+ High Disturbance. Anthropogenic disturbance changed the availability of some classes of microhabitat, most notably the removal of loose rocks, reduction in woody plants in conversion for grazing and agriculture, creation of pools on some low-level sites and importation of soil at Panchgani (Fig 2).

**Table 1:** Disturbance values and dominant land use for each site surveyed. To facilitate spatial comment, the study area has been sub divided into three latitudinal zones each side of the escarpment.

Site	Latitude	Longitude	Land use	Disturbance intensity	Eco-zone
Chalkewadi	17.5736	73.8261	Wind turbine	Medium	High North
Jagmin	17.5927	73.8181	Natural grazing	Low	High North
Mhavashi	17.4310	73.9313	Wind turbine	Medium	High North
Panchgani	17.9217	73.8045	Tourism	High	High North
Masai	16.8181	74.0779	Tourism/grazing	High	High Central
Zenda	16.9226	73.8072	Natural grazing	Low	High Central
Amboli Low	15.9374	74.0027	Tourism	High	High South
Amboli High	15.8903	74.0403	Natural grazing	Low	High South
Shipole	17.9735	73.0527	Agriculture	Low	Low North
Ratnagiri	16.9627	73.2962	Agriculture	Medium	Low Central
Lanja	16.7419	73.4204	Natural grazing	Low	Low Central
Kudopi	16.2327	73.5105	Natural grazing	Low	Low South
Dhamapur	16.0315	73.584	Agriculture	Medium	Low South

### Field data collection methodology

Sampling of both microhabitat and amphibian presence was performed along the same belt transects concurrently. The rocky plateaus are relatively simple ecosystems dominated by areas of exposed rocks with varying amounts of other microhabitats. Their size varies by an order of magnitude but based upon the smaller sites within the survey it was determined that four belt transects each 100 m long and 6 m wide would adequately encompass all the microhabitat types available on an individual plateau. The direction and path of each transect was determined at each site to maximise sampling of all available microhabitats. The same methodology was applied on each survey on the same plateaus in each year but with different transect locations making a total surveyed area 4800m^2^.

To maximise detection, both diurnal and nocturnal surveys were deployed during two temporally comparable survey seasons [70]. Surveying took place each year in the same weeks at the onset of the monsoons in late July to early August in 2013 and 2014 [71]. Survey timing was selected for the known range of amphibian autecology, encompassing taxa with both explosive and prolonged breeding strategies [36, 72].

To make samples comparable, standardised Visual Encounter Surveys (VES) with refugia searching [67] along the belt transects were performed [73, 74]. The identity of each amphibian taxa their abundance and their microhabitat associations were recorded for each section of the transect [75]. Where species identity was not immediately obvious in the field photographs were taken to permit later clarification.

Microhabitat variables recorded along the same transects as the VES surveys comprised; maximum refugia rock size (mm), number of loose rocks >50mm, woody plant cover (as % cover on transect), presence of soil depressions with vegetation, presence of flowing streams, presence of static pools, presence of surface flooding (vernal pools). Although some microhabitats co-occurred, e.g. surface flooding and stream presence, all were included in the analysis so that finer scale associations could be detected (Fig 2). As some NWG amphibians are semi-terrestrial humidity levels may be considered as a micro-habitat therefore Relative humidity included in the analysis, it was measured with a calibrated hygrometer (Hanna Instruments™ HI 9064; [76].

All amphibians were identified using the best available literature, and their nomenclature considered using the latest taxonomical authorities [6, 26, 69, 77–85]. The classification of several of the taxa found in this study is still evolving. While many herpetologists have adopted the new suggested taxonomies entirely, this study adhered to recommendations within [86] and [87] by presenting former nomenclature alongside more recent identifications to maintain the continuity of identification in years following taxonomic amendments. This system introduces new and unstable taxa with the formerly acknowledged genera first and the newly identified genera in parentheses. For example, although the changes proposed by Frost [88] for the genus *Rana* were made at the generic level, biologists wishing to recognize the subdivisions of this genus, but maintain the stability of familiar species names and still follow rules of the International Code of Zoological Nomenclature (ICZN), can recognise newly created subdivisions of these genera as subgenera [86, 87, 89]. Under ICZN rules, the subgenus category may follow the genus name in parentheses, e.g., *Fejervarya* (*Minervarya*) *sahyadris* or *Rana* (*Lithobates*) *pipiens*.

### Statistical analytical methods

Primer-e and Permanova+, Primer-e v7 [90, 91] were used to investigate the relationships between taxa in the study area and their microhabitats. Biotic data were represented by a Bray-Curtis similarity matrix of square root transformed abundance. Environmental data were represented by a Euclidian Distance matrix which was normalised before analysis. Analyses were performed for all taxa combined and each individual taxon. Ordination and visualisation of the model was performed in distance-based redundancy analysis (dbRDA). To identify the microhabitats with significant taxa associations’ step-wise analysis was performed in distLM. The step-wise routine commences with a null model then adds each criterion before checking by tentative removal thus optimising the selection. As the sample and number of predictor variables were small the Akaike Information Criterion with second order correction (AICc), was used as it to accounts for the ratio of samples to predictor variables being lower than 40 and performed in distLM [91, 92]. The explanatory power of microhabitats for the distribution of the biota was assessed using LINKTREE, a form of constrained binary divisive clustering. The routine maximises the value of Rat each division in the biotic matrix in concordance with the underlying distribution of microhabitats within each patch (site) with the B% being the difference in each linkage [90, 93].

### Ethics statement

Sampling was undertaken by kind permission of the Indian National Biodiversity Authority, Chenai, India under permit number: Maharashtra 2014 MC200621.

The advice from the representative of the University of Plymouth’s Animal Welfare and Ethics Committee was that no formal consent was required since the animals were only observed or received minimal handling on their site of origin. We followed strict handling and preventative measures for cross-contamination, following standard practice for working with amphibians as described on http://www.amphibiaweb.org. No endangered animals were specifically targeted in the study.

## Results

A total of 325 individual amphibians from 2 orders, 6 families, and 21 taxa were detected over the two years of study (S1 Table). Abundance, taxa and microhabitats varied between all sites. Only 47% of recorded microhabitat associations were in accordance with the IUCN habitat descriptions (Tables 2 & 3; [9]). The 21 taxa in the study represent a small proportion of the known amphibian taxa from India (419 from India [26] and 161 from Western Ghats [25]) but almost 40% of those that are known to occur in Maharashtra [53; 94]. Distribution data can be accessed in the Supporting Information.

**Table 2:** Habitat association results from significant habitat associations identified in step wise analysis using AICc in distLM, Permanova+, Primer-e v7, where * = P<0.05, ** = P<0.01.Status is the IUCN threat status: Accessed 10/02/2017 [9]. NA- Not Assessed; DD-Data Deficient; LC-Least Concern; EN-Endangered; CR-Critically Endangered. Population stability:/S-Stable; /D-Decreasing; /I-Increasing. RH-Relative Humidity; Rock -large loose rocks >50 mm; Rock N-abundance of small rocks<50 mm; Plant-%of area with woody plant cover; Soil-% of area with soil; Stream-stream in surveyed area; Pool-lentic pools within surveyed area; Flood-plateau surface flooded to a depth >25 mm; Agree-our habitat association agree with published findings; Elev-altitude above sea leavel taxa were found; Habitat Associations are those listed by the IUCN.

Taxa	Status	RH	Rock	Rock N	Plant	Soil		Stream	Pool	Flood	Agree	Elev	Habitat Associations
*All taxa combined*		**	* *	* *	**	* *	* *		**	**		*0–1179*	
*Duttaphrynus melanostictus*	*LC/I*	* *	* *	* *	*		*		*	*0*.*09*	*x*	*809–1131*	Generalist
*Euphlyctis cyanophlyctis*	*LC/S*	* *	* *	* *	* *	* *	*		* *	* *	*x*	*85–1131*	Lentic, ephemeral water, forest, shrubland
*Fejervarya* (*Zakerana*) cf. *caperata*	*DD*	* *	* *	* *	* *	* *	*0*.*07*		* *	* *	*x*	*1156–1090*	Semi-aquatic, grassland, plateaus, disturbance tolerant
*Fejervarya* (*Zakerana*) *cepfi*	*NA*	*0*.*08*	* *	* *	* *	*	* *		* *	**		*85–156*	Degraded forest
*Gegeneophis* cf. *ramaswamii*	*LC/S*	* *	* *	* *	* *	*0*.*08*	* *		* *	* *	*x*	*809*	Generalist, fossorial.
*Gegeneophis seshachari*	*DD*	* *	* *	**	* *	*	* *		*	**		*90–156*	Forest, plantations, gardens, degraded forest
*Hoplobatrachus tigerinus*	*LC/S*	**	* *	* *	**	* *	*		**	*	*x*	*67–1131*	Generalist very adaptable
*Indirana chiravesi*	*LC/D*	* *		*	* *	* *	* *		*	* *	*x*	*1015*	Aquatic, lotic
*Indotyphlus maharashtraensis*	*DD*	* *	*	* *	*	* *	**		* *	* *	*x*	*1179*	Dry grassland
*Microhyla ornata*	*LC*	* *	* *	**	* *	*	* *		*	*0*.*06*	*x*	*85–170*	Savanna, shrubland, grassland, lentic, lotic
*Fejervarya (Minervarya) sahyadris*	*EN/D*	*	* *	*	* *	* *	* *		**	**		*85–170*	Grassland, pasture, seasonal flooding, lentic
*Xanthophryne tigerina*	*CR/D*	*0*.*08*	**	* *	* *	* *	* *		* *	*	*x*	*809–854*	Lateritic plateaus

**Table 3:** The most important microhabitats for taxa that tested without significant habitat associations in the AICc analysis in distLM Permanova+, Primer-e v7. Status is the IUCN threat status. Accessed 10/02/2017 [9]. NA- Not Assessed; DD-Data Deficient; LC-Least Concern; EN-Endangered; CR-Critically Endangered. Population stability:/S-Stable; /D-Decreasing; /I-Increasing. RH-Relative Humidity; Rock -large loose rocks >50 mm; Rock N-abundance of small rocks<50 mm; Plant-%of area with woody plant cover; Soil-% of area with soil; Stream-stream in surveyed area; Pool-lentic pools within surveyed area; Flood-plateau surface flooded to a depth >25 mm; Agree-our habitat association agree with published findings; Elev-altitude above sea leavel taxa were found; Habitat Associations are those listed by the IUCN.

Taxa	Status	RH	Rock	Rock N	Plant	Soil	Stream	Pool	Flood	Agree	Elev	Habitat Associations
*Fejervarya* (*Zakerana*) cf. *brevipalmata*	*DD*	* *				*x*			*x*	*x*	*1131–1157*	Forest, grassland, wetland, degraded forest
*Fejervarya* sp.		* *			*x*						*1090*	
*Indotyphlus* cf. *battersbyi*	*DD*	* *	*x*								*974*	Forest, shrubland, plantations, gardens, degraded forest
*Philautus* sp.		* x*									*170*	
*Polypedates maculatus*	*LC/S*	* *							*x*		*156*	Forest, shrubland, lentic, disturbance tolerant
*Pseudophilautus* sp.		* x*									*170*	Forest, degraded forest.
*Raorchestes* g*hatei*	*NA*	* *							*x*		*1131–1179*	
*Sphaerotheca dobsonii*	*LC/D*	* *				*x*					*85–974*	Lowland forest, shrubland, seasonal lentic
*Uperodon globulosus*	*LC/S*	*0*.*07*	* *	* *	* *	* *	* *	* *	* *		*67*	Generalist, anthropogenic environments, disturbance tolerant, generalist

### Spatial distribution of microhabitats

**Fig 3:**
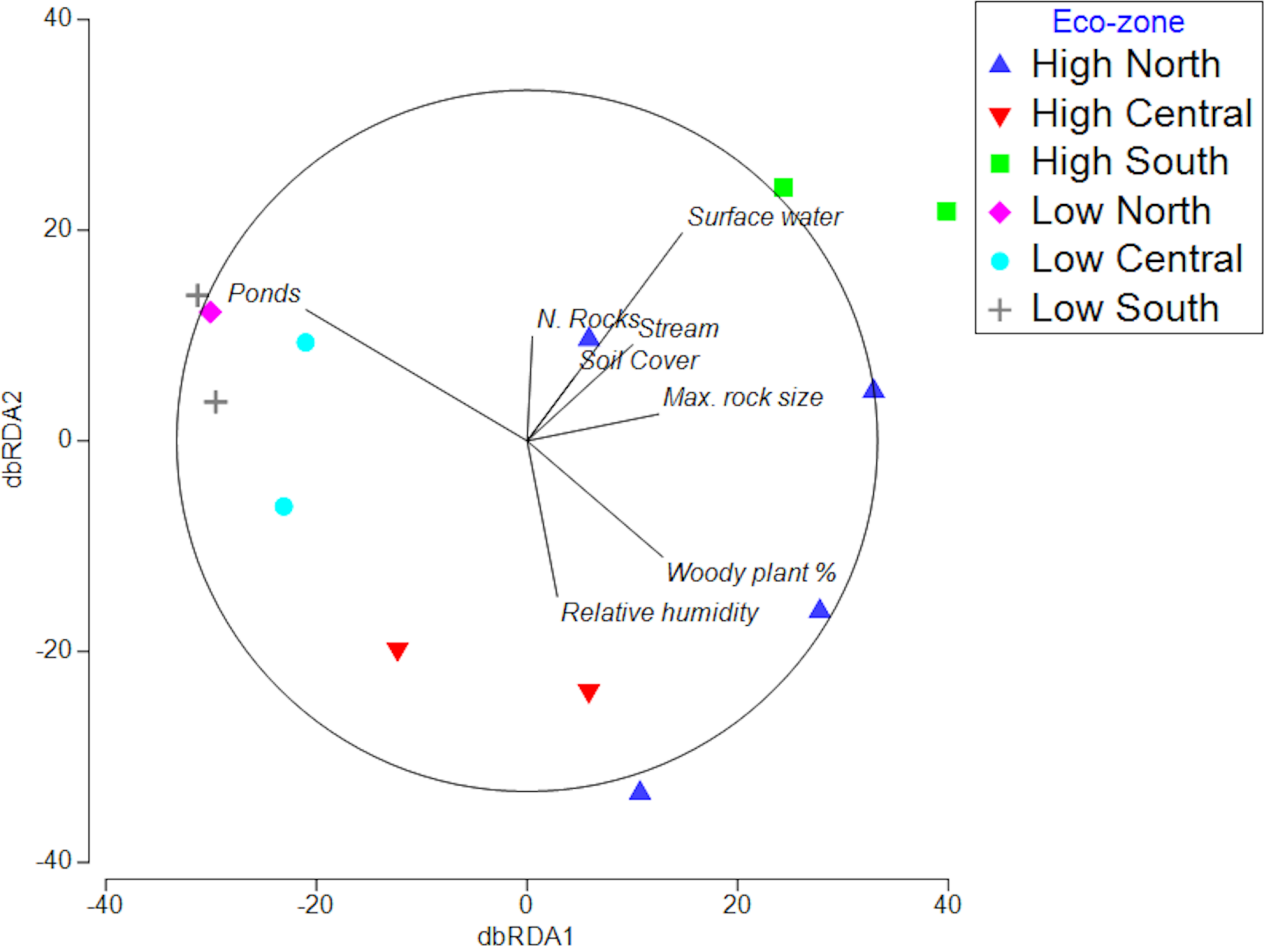
dbRDA analysis for microhabitats with sites illustrated within eco-zones to allow spatial comparison. dbRDA1 explained 39.3% of fitted data and 25% of total variation with dbRDA2 explaining 19.3% of fitted data and 12.3% of total variation.

**Fig 4:**
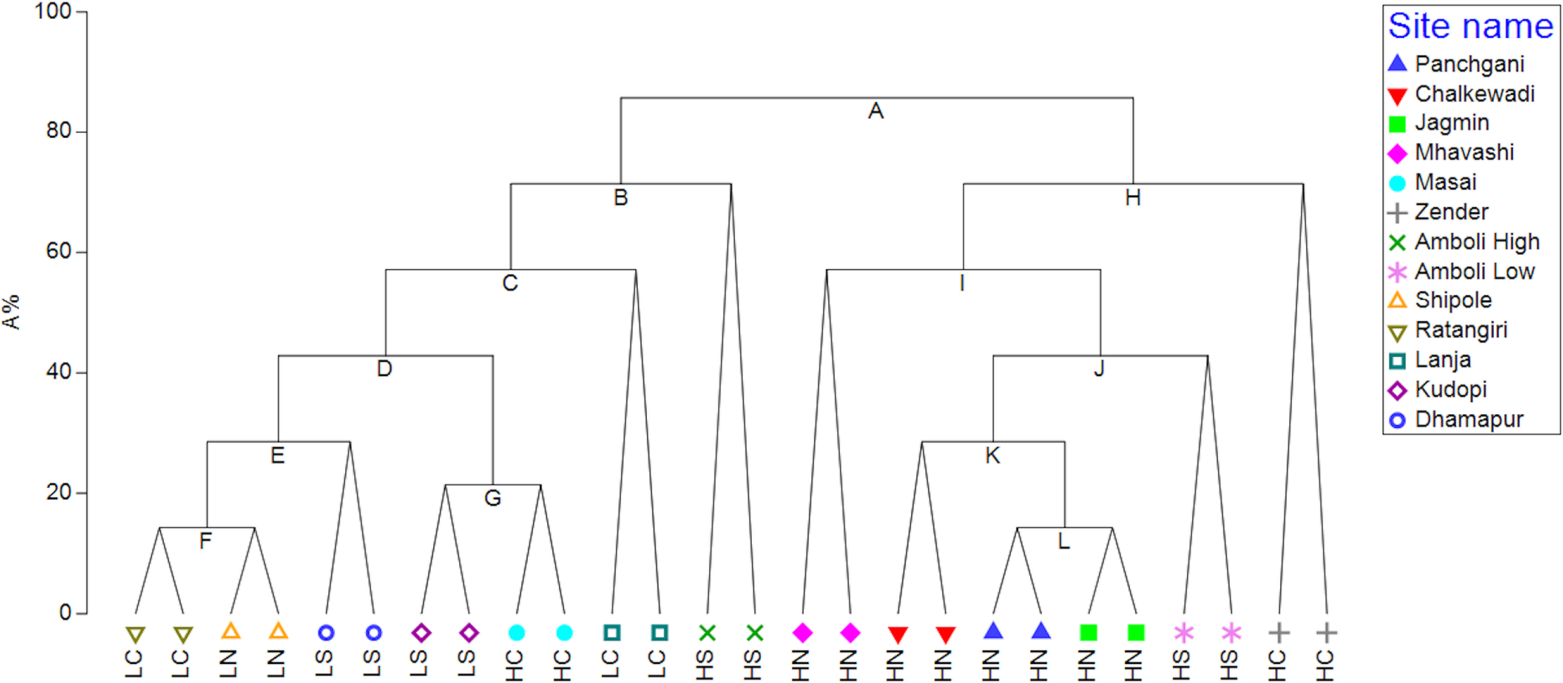
Linktree analysis of plateau similarities based upon microhabitat explanations for the biotic distribution, Primer-e v7. Annotated for Eco-zones; HS-High South; HC-High Central; HN-High North; LS-Low South; LC-Low Central; LN-Low North. A: R = 0.53; B% = 85; Woody plants<-0.117(>-0.0826). B: R = 0.89; B% = 91; Max loose rock size<0.832(>2.26) or Surface water<0.848(>1.67) or Woody plants>-0.999(<-1.23). C: R = 0.55; B% = 43; Surface water>-1.19(<-1.6) or Woody plants<-0.345(>-0.117). D: R = 0.37; B% = 29; Woody plants<-0.738(>-0.607). E: R = 0.54; B% = 20; Stream>0.743(<-1.1) or Max loose rock size<-0.871(>0.00354) or Surface water>-0.377(<-0.785) or Woody plants>-0.738(<-0.999). F: R = 0.00; B% = 11; Max loose rock size<0.00354(>0.832) or Soil Cover<-0.791(>0) or N. Rocks>-0.0943(<-0.7) or Surface water>-0.785(<-1.19). G: R = 1.00; B% = 26; N. Rocks<-1.09(>2.13) or Pools<-0.832(>1.23) or Surface water>0.848(<-1.19) or Soil Cover<0(>1.58) or Max loose rock size<-1.42(>-0.0425) or Stream>0.743(<-0.177) or Woody plants<-0.607(>-0.345). H: R = 0.37; B% = 68; N. Rocks<-0.067(>1.63) or Woody plants>0.376(<-0.0826). I: R = 0.54; B% = 67; Woody plants>2.31(<1.39) or N. Rocks<-1.03(>-0.997). J: R = 0.63; B% = 55; Soil Cover<0(>2.37) or N. Rocks<-0.206(>-0.067). K: R = 0.50; B% = 42; Surface water<-0.377(>0.44). L: R = 0.50; B% = 23; Stream<-1.1(>1.66) or Max loose rock size<-1.24(>0.786) or Woody plants<0.376(>1.39) or Surface water>1.26(<0.44) or Soil Cover<-0.791(>0) or N. Rocks<-0.997(>-0.206) or Pools<-0.832(>-0.317).

Sites could be spatially separated at the macroscale by the relative microhabitat composition with notable differences above and below the escarpment illustrated in Figs 3 and 4 (Fig 4; R = 0.53, B% = 85%; Fig 3). The two most distinctive sites, Amboli High (Fig 4; R = 0.89, B% = 91) and Zenda (Fig 4; R = 0.37, B% = 68), are low disturbance sites that have retained much of their loose rock cover and have taxa associated with rock refugia (Fig 3). Lanja, a low disturbance site, is the most charismatic of the low region sites (Fig 4; R = 0.55, B% = 43; Fig 2). The most diverse eco-zone was the High North as illustrated by the distribution of the data points in the dbRDA plot, reflecting the impact of three types of land use on microhabitat availability (Fig 3, Table 1).

Pools were more abundant below the escarpment where many are manmade; their hydroperiod is shorter on the northern sites and more consistent above the escarpment. The number of annual wet days declines south to north by 11% and there 12.7 times as many wet days above the escarpment (Fig 1; [69–96]). Rainfall amount peaks at Amboli where it exceeds 9000 mm per annum resulting in the microhabitat ‘surface water’ separating the two Amboli sites from the rest (Fig 3). Soil is scant on the plateaus but deepens where it has accumulated in shallow depressions in the ferricrete but it has also has been imported onto Panchgani plateau to assist in tourism related activity [12]. Loose rock abundance, important as refugia, breeding sites and mate advertising posts, was reduced by collection from accessible sites for construction resulting in a disturbed distribution pattern (Fig 1; Tables 2 & 3; [12, 13, 48, 97]). Larger loose rocks were most absent from plateaus below the escarpment and most abundant where human access was difficult for example in the remoter High Region plateaus for example Zenda and Amboli High and to a lesser extent Jagmin. (Fig 2; Fig 4B, 4H & 4L; [98–100]). That, combined with greater woody plant cover, and for some sites surface water, separated them from the low sites and explained much of the latitudinal divisions (Figs 3 & 4A, 4B, 4H, 4I, 4J and 4K).

Most sites were characterised by combinations of microhabitats and their associated taxa (Fig 3; Tables 2 & 3). Such combinations are key for some taxa for example soil and rocks used by caecilians as refugia and egg deposition sites associated with soil close to water. We found 56% of microhabitats to be impacted by anthropogenic activity Tables 2 & 3).

### Spatial distribution of taxa and explanatory microhabitats

Pond presence on low elevation sites was the most significant abiotic variable separating their amphibian assemblages from those above the escarpment where woody plants, surface water and relative humidity were the principal characters (Figs 3 & 4). Woody plant abundance, maximum loose rock size, surface water and pond presence were significant factors defining the differences in the biota above and below the escarpment in both the dbRDA and LINKTREE analyses (Fig 3; Fig 4, 4A, 4B & 4D).

High-level sites had 9 taxa not found on low sites (Figs 2 & 3; Tables 2 & 3). Woody plants were significantly associated with 44% of exclusive high-level taxa with no such associations for low-level specialists (Tables 2 & 3). Of the 8-taxa found exclusively on low sites 63% had significant relationships with water bodies and 60% of those were associated with pools compared to only 22% on high-level sites. However, all 8 had a relationship with the co-occurring surface flooding, highlighting the need to carefully define the types of water body.

Twelve of the 21-study taxa had significant habitat associations with the remaining 9 having associations that, whilst not significant, were identifiable (Tables 2 & 3). Taxa in the study are described as generalists after the IUCN description where they lack habitat specificity. Generalists that are very widely distributed indicating broad climatic and habitat tolerances are described as ubiquitous [9]. The remaining taxa, *Uperodon globulosus*, characterised as a generalist, was only detected when it was raining and had a relationship with relative humidity perhaps explaining its limited detection (Table 3 [9]). The generalist taxa did not have a noticeably higher number of habitat associations than other taxa except *Hoplobatrachus tigerinus*, which whilst currently described as a generalist, should more appropriately be assessed as ubiquitous.

Some 52% taxa were found in habitats other than those recorded by the IUCN, with 91% of the taxa sampled not previously recorded from lateritic plateaus [9]. Just over 67% of taxa in the study were associated with water bodies. Surface Flooding was the most important form of water on the plateaus being significant for 48% of taxa, Pools for 33% and Streams for 24%. Of the pool specialists 50% were only found on low level sites where pools were more common. Loose rocks measured by both size and abundance were the next most important microhabitat being significant for 33% of taxa, 19% with small rock abundance and 14% associated with large rocks. Rock sizes and abundance were meaningful in defining the different regions where large rocks were important for 33% of high-level specialists (Tables 2 & 3). The abundance of small rocks (<50 mm) was essential for 33% of exclusively low-level taxa but only one high-level specialist (Tables 2 & 3).

There were 29% of taxa associated with soil-filled depressions. Only 19% of taxa were associated with woody plants despite their being one of the defining microhabitats (Fig 2, Tables 2 & 3). The IUCN lists just 2 of the 21 taxa found as being associated with lateritic plateaus [9]. Generalist taxa were associated with a higher number of microhabitats (mean 3.7) than the other taxa (1.7). *Fejervarya* (*Zakerana*) cf. *cepfi*, *Raorchestes* cf. g*hatei* have not been assessed yet and their association should be noted with their first assessment. *Fejervarya* (*Zakerana*) cf. *caperata*, *Gegeneophis seshachari*, *Indotyphlus maharashtraensis* and *Indotyphlus* cf. *battersbyi* are all data deficient, thus the data presented here will form part of their initial assessment.

Many amphibians were detected under lateritic rock refugia in diurnal surveys. Only 5 taxa, *Hoplobatrachus tigerinus*, *Fejevarya* cf. *caperata*, *Fejevarya* (*Minevarya*) cf. *sahyadris*, *Indotyplus* cf. *beddomii* and *Xanthophryne tigerina* were found across open areas during the day, and these were only encountered during rainy periods. The above open area taxa were often well camouflaged against the texture of the lateritic rock or among short grass growing on soil depressions. Nocturnal transects confirmed the presence of most of the diurnal anurans without adding new taxa to the sample.

### Caecilian microhabitats on lateritic plateaus

Soil is important for many amphibians providing sites to aestivate but is critical the semi-fossorial caecilians [101–103]. Three of the 4 caecilian taxa were associated with rocks in addition to areas of soil or stream presence (Tables 2 & 3). The exception was *Gegeneophis* cf. *ramaswamii*, considered a generalist fossorial taxon, a view this study supports from results associating it with soil-filled depressions (Table 2; [9]). We observed that *Indotyphlidae* sp, were detected diurnally under lateritic rocks that were positioned on soil depressions indicating the importance of co-occurrence of some microhabitats. These soil depressions were often no deeper than 10 cm. *Gegeneophis* cf. *ramaswamii*, *G*. *seshachari*, *Indotyphlus* cf. *battersbyi* and *I*. *maharashtraensis* were all located between the rock and the soil substrate although not significantly for *G*. cf. *ramaswamii*. One single *I*. *maharashtraensis* at Jagmin was found emerging from a soil depression next to rain fed flowing run-off stream after nearby terrain was disturbed by searching activity. The rocks caecilians were detected under were all within a short distance (no more than 20 m) from surface run-off, stream, or wet seep areas supporting the view soil moisture was likely to be highly important to the group [104]. The *Gegenophis* sp are oviparous and use rocks to shelter their young, for example, *Gegeneophis seshachari* at Kudopi comprised a mixture of adult and juveniles all found under rocks within a single 50m stretch of wet run-off [80].

## Discussion

Much literature only describes the broad habitat and not the microhabitats required by the individual taxa for example forest or savannah [9]. The distribution of microhabitats on the plateaus in the NWG was non-random irrespective of the scale of observation as their pattern reflects the edaphic processes, macroclimate and disturbance factors at play in the region (Figs 2, 3 & 4). The presence and abundance of some of those microhabitats were changed by human activity. All taxa in the study had identifiable habitat associations, with the majority being significant (Tables 2 & 3). The study found that each lateritic plateau, whilst having core microhabitat similarities, had a unique habitat and thus identity. Therefore, a macroscale distribution amphibian pattern derived from macroclimate and surrounding countryside alone was imperfect and patch quality in terms of microhabitat availability and thus regulating patch habitat must be included as explanatory factor. We find patch quality, within a climatic region, was best defined by its microhabitat mix. Some microhabitat availability was directly related to anthropogenic activity. The rarest taxa in the study were the most sensitive to anthropogenic habitat alteration.

The plateaus have localised microclimates and offer habitats, comprised of mosaics of microhabitats, and are at high ecological contrast to the surrounding landscape [12]. There is evidence to suggest that has resulted in genetic isolation between plateaus in other taxa [105, 106]. The resulting amphibian distribution reflects both the isolation and divergent pressure within the WG through the exceptional levels of endemism on the plateaus of the NWG; 61% of the sample were endemic to Asia, 52% to India and 38% to the WG with *Raorchestes* g*hatei* and *Xanthophryne tigerina* only known from lateritic plateaus (Tables 2 & 3). More common taxa, which we characterise as generalists, are able to move through the countryside between plateaus and can persist on plateaus through metapopulation dynamics [13, 65, 107]. Both the common taxa and the rare ones that cannot cross the space between plateaus are reliant upon suitable habitat availability within each plateau [12, 13, 64]. Therefore, habitat quality was highly important in determining the presence and persistence of many taxa but most importantly the rare ones. That quality depends on both landscape level variables including climate, seasonality and topography and within-plateau elements [63].

Many amphibians use water as their primary habitat to avoid desiccation or predation and as a breeding resource and that was reflected with the majority (67%) of the sample being associated with water bodies, a figure very close to that published for other areas in the WG (62%), (Tables 2 & 3; [25, 43, 102]). However, non-aquatic microhabitat associations were also found for 78% of the sample taxa (Tables 2 & 3). Those microhabitats, climatic and habitat combinations fulfil a variety of ecological purposes beyond their basic physiological requirements; refugia from climatic extremes [108] and predators [109, 110], mate advertisement perches [48], sites for egg deposition [111, 112], breeding resources [36, 43, 113]; reproductive behaviour is selected for by suitable rainfall and relative humidity conditions [48, 106, 114].

### Seasonal changes in microhabitat use

Many of the plateau taxa breed close to the start of the monsoon and they may have been detected in association with their breeding microhabitats [48, 115]. The plateaus are all highly seasonal only receiving rainfall for around four months a year resulting in the need for seasonal movement to avoid desiccation and to access breeding sites [36, 106, 115]. Rainfall, hydroperiod and the associated relative humidity are important factors for taxa with terrestrial or semi-terrestrial larvae for example *Xanthophryne tigerina* which was found only in the very wet southern high sites [6, 36, 48].

### Generalist taxa microhabitat associations

Generalist taxa in this study were associated with more than twice as many types of microhabitat than the mean for other taxa (Table 2). However, the IUCN definition may be spatially too coarse to adequately describe patch quality as it makes little reference to microhabitat associations. There were two non-generalist taxa, *Gegeneophis seshachari* and *Microhyla ornata*, with very similar number of associated microhabitats (4) to the generalist total (3.7) suggesting that they too were generalists. However, such a result can be explained by co-occurrence microhabitats necessary for some taxa. For example, the microhabitats for *Gegeneophis seshachari* encompass a range predictable for a caecilian; rock, soil and water (Table 2). Another, *Gegeneophis* cf. *ramaswamii*, was perhaps wrongly identified as a generalist as it appears to require specific combinations of microhabitats to persist but can also be found among a range of landscapes. Similarly, *Microhyla ornata* should be reclassified as a habitat generalist in the context of lateritic plateaus. The generalist taxa, *Duttaphrynus melanostictus* and *Hoplobatrachus tigerinus* each have associations with all three aquatic microhabitats. This was an unsurprising result as both are pond breeding taxa that are also associated with abundant woody plant cover (Table 2; [32, 33]).

### Impact of elevation on microhabitat associations

Tropical site habitats are known to change with elevation a view supported by this study (Figs 2 & 3; [41, 42, 116]. The drivers of elevational differences in the amphibian assemblages on the plateaus of the NWG were microhabitats dependent upon rainfall increasing which increased in frequency and volume with increasing elevation and hydroperiod which decreases with latitude. Although not directly related to elevation the ease of access onto low elevation sites, and their agricultural land use, has increased man-made pool frequency and reduced the abundance of large rocks (Figs 2, 3 & 4). The combination of long periods of rainfall, the related high relative humidity and abundance of loose rocks on Amboli High and to a lesser extent Amboli Low creates a special habitat the critically endangered and declining Amboli Toad, *Xanthophryne tigerina*, The large rocks provide three major resources, refugia, breeding sites and mate advertisement sites [48]. All of these are highly important resources for not only *X*. *tigerina*, *but* as breeding sites for Caecilians [117]. Woody plant abundance was one of the main microhabitats to define the regional difference between the high and low-level sites (Figs 3 & 4). Together with its associated soil filled depressions woody plants were highly important in amphibian distribution on the NWG plateaus across all elevations but impacting different taxa (Tables 2 & 3).

### The effect of anthropogenic disturbance on amphibian microhabitats

Microhabitat availability was changed by three forms of anthropogenic disturbance on the plateaus; removal e.g. loose rocks, damage e.g. trampling or cutting down of plants and alteration by addition of foreign material e.g. soil at Panchami. Anthropogenic disturbance was also evidenced by construction and pollution. We did not examine the impact of addition by construction, pollution or trampling and therefore cannot comment specifically on these, although the sites with wind turbines had some construction on them. All rural communities close to the plateaus carried out the common practice of harvesting loose rock and utilising it for construction of dwellings, walls and memorials [12]. Therefore, sites at which there were quantities of rocks >50 mm^3^ were often farther from human residences. Given that many of the amphibian taxa in this study were associated with, detected under, or proximate to cover provided by rocks >50 mm^3^ we suggest that the natural occurrence of rocks >50 mm^3^ on plateau sites is an essential microhabitat resource for all amphibians, and one that is a rapidly emerging conservation concern for all plateaus.

Disturbance by the addition of soil, together with tourist related activity; on the high-level site Panchgani has shifted the taxa assemblage towards one dominated by generalist or widely distributed taxa (Tables 2 & 3). The addition of soil has closed almost all the fissure refugia and all large loose rocks and most small ones have been removed, limiting the available types of refugia, breeding and mate advertisement sites. This site is popular with equine tourism and this local industry has resulted in infrastructure development (cafes, stables and roads), soil compaction and increasing levels of domestic refuse. The pools also have a high silt load from the imported soil and grazing. A total of 24 individual amphibians were recorded from this plateau. Although amphibian counts were relatively high in comparison to lower disturbance sites, several of the taxa recorded (*D*. *melanostictus*, *E*. *cyanophlyctis* and *H*. *tigerinus*) are considered widespread or generalist taxa, listed as ''least concern'' in the IUCN status reports (Tables 2 & 3). *D*. *melanostictus*, *E*. *cyanophlyctis* and *H*. *tigerinus* were anticipated as taxa known to associate with anthropocentrically disturbed or modified habitats (Daniel, 2002). However, the presence of *Raorchestes* cf. *ghatei* and *Fejevarya* cf. *brevipalmata* may be surprising as they had limited distribution and are data deficient taxa in need of more robust ecological and population studies (Tables 2 & 3). Panchgani has a number of large pools constructed for watering livestock and anthropogenic uses. The largest is likely to hold some water throughout an average year possibly shaping the community by offering aquatic refugia in the dry season not seen on many sites. That may be a significant factor structuring the assemblage as it would favour pond specialist taxa [118].

Surface topography on the low-level plateaus creates some pools but many additional ones have been created by farmers in association with their agricultural land use. At a landscape level pool frequency is important in maintaining population connectivity and persistence [118–120].

### Impact of climate change on the amphibians of the northern Western Ghats

Two changes in climate are predicted to impact the amphibian microhabitat requirements in the NWG: increasing temperature and fragmentation of the monsoon rains [121]. Both will require microhabitat resources to mitigate their effects; as refugia from increased temperatures and desiccation [107, 122]. The woody plants and rocks in this study provide thermal refugia allowing behavioural temperature regulation and are therefore key microhabitats worthy of preservation [122]. Breaks in rainfall that occur when larvae are in pools or in hygropetric habitats are likely to cause significant losses. To offset these, maximum availabilities of both populations and microhabitats should be preserved.

## Conclusion

We conclude that microhabitat availability is a good way of defining patch quality for amphibians within a climatic zone and preserving patch quality is important for conserving amphibians. The study, as the first statistically supported in the NWG, has added substantially to known amphibian microhabitat associations. Spatial variation in microhabitat distribution in part explains amphibian diversity on the threatened lateritic plateaus in the NWG. The preservation of a wide a range of microhabitats is clearly important for amphibian conservation. It is clear that the NWG lateritic plateaus, with their unique microhabitat assemblages, are highly important habitats for a significant number of threatened amphibians and conservation policy should aim to preserve representative plateaus from each eco-zone. Preservation of microhabitats that provide thermal and desiccation refugia will become increasingly important for the persistence of plateau amphibians in the face of increasing temperatures and a more fragmented monsoon; these include pools, large rocks and woody plants [121].

## Supporting information

S1 Table Breakdown of abundance data by taxa, site, eco-zone, year and day-night time survey. (DOCX)

## References

[1] Myers N, Mittermeier RA, Mittermeier CG, da Fonseca GAB, Kent J. Biodiversity hotspots for conservation priorities. Nature. 2000;403(6772):853–8. doi: 10.1038/35002501 10706275 10.1038/35002501

[2] Sloan S, Jenkins CN, Joppa LN, Gaveau DLA, Laurance WF. Remaining natural vegetation in the global biodiversity hotspots. Biological Conservation. 2014;177:12–24.

[3] Cincotta RP, Wisnewski J, Engelman R. Human population in the biodiversity hotspots. Nature. 2000;404(6781):990–2. doi: 10.1038/35010105 10801126 10.1038/35010105

[4] Widowson M, Cox K.G. Uplift and erosional history of the deccan traps India: Evidence from laterites and drainage patterns of the Western Ghats and Konkan coast. Earth and Planetary Science Letters. 1996;137:57–69.

[5] Ram MS, Marne M, Gaur A, Kumara HN, Singh M, Kumar A, et al Pre-Historic and Recent Vicariance Events Shape Genetic Structure and Diversity in Endangered Lion-Tailed Macaque in the Western Ghats: Implications for Conservation. Plos One. 2015;10(11):e0142597 doi: 10.1371/journal.pone.0142597 26561307 10.1371/journal.pone.0142597 PMC4641736

[6] Biju S, Van Bocxlaer I, Giri V, Loader S, Bossuyt F. Two new endemic genera and a new species of toad (Anura: Bufonidae) from the Western Ghats of India. BMC Research Notes. 2009;2(1):241. 19968866 10.1186/1756-0500-2-241 PMC2797014

[7] Vidya T, Fernando P, Melnick D, Sukumar R. Population differentiation within and among Asian elephant (Elephas maximus) populations in southern India. Heredity. 2005;94(1):71–80. doi: 10.1038/sj.hdy.6800568 15454948 10.1038/sj.hdy.6800568

[8] Padhye AD, Ghate HV. An overview of amphibian fauna of Maharashtra State. Zoo's Print Journal. 2002;17:735–40.

[9] IUCN. The IUCN Red List of Threatened Species 2016–2. Available from: http://www.iucnredlist.org/search. 10.3897/BDJ.4.e10356 PMC5018116 27660524

[10] Widdowson M. Laterite and Ferricrete In: Nash DJ, McLaren S.J., editors. Geochmeical Sediments and Landscapes. Oxford, UK: Wiley-Blackwell; 2007 pp. 46–94.

[11] Porembski S, Watve A. Remarks on the species composition of ephemeral flush communities on paleotropical rock outcrops. Phytocoenologia, 35. 2005;2(3):389–402.

[12] Watve A. Status review of Rocky plateaus in the northern Western Ghats and Konkan region of Maharashtra, India with recommendations for conservation and management. Journal of Threatened taxa. 2013;5(5):3935–62.

[13] Thorpe C, Watve A. Lateritic Plateaus in the Northern Western Ghats, India; a Review of Bauxite Mining Restoration Practices. Journal of Ecological Society, Pune, Maharashtra, India. 2016:25–44.

[14] Lekhak M, Yadav SR. Herbaceous vegetation of threatened high altitude lateritic plateau ecosystems of Western Ghats, southwestern Maharashtra, India. Rheedea. 2012;22(1):39–61.

[15] Porembski S, Silveira FAO, Fieldler PL, Watve A, Rabarimanarivo M, Kouame F, et al Worldwide destruction of inselbergs and related rock outcrops threatens a unique ecosystem. Biodiversity Conservation. 2016; Letter to the editor.

[16] Pinder A, Halse S, Shiel R, McRae J. Granite outcrop pools in south-western Australia: foci of diversification and refugia for aquatic invertebrates. Journal of the Royal Society of Western Australia. 2000;83(3):149–61.

[17] Jocqué M, Vanschoenwinkel B. and Brandonck L. Freshwater rock pools: a review of habitat characteristics, faunal diversity and conservation value. Freshwater Biology. 2010;2010:1–16.

[18] Hopper SD, Silveira FA, Fiedler PL. Biodiversity hotspots and Ocbil theory. Plant and Soil. 2015:1–50.

[19] Bharucha EK. Current ecological status and identification of potential ecologically sensitive areas in the Northern Western Ghats. Pune, Maharashtra, India: Bharti Vidyapeeth Deemed University, Research IoEEa; 2010 Available from: www.moef.nic.in/downloads/public-information/Annexure5-7th.pdf.

[20] CEPF. Asia-Pacific Biodiversity Hotspots: Critical Ecosystem Partnership Fund; 2016.cited 2016. Available from: http://www.cepf.net/resources/hotspots/Asia-Pacific/Pages/default.aspx.

[21] Balaji D, Sreekar R, Rao S. Drivers of reptile and amphibian assemblages outside the protected areas of Western Ghats, India. Journal for Nature Conservation. 2014;22(4):337–41.

[22] Lad RaS J.,S. Environmental impact of Bauxite mining in the Western Ghats in south Maharashtra, India. International Journal of Recent Scientific Research. 2013;4(8):1275–81.

[23] Phillips J. Using a mathematical model to assess the sustainability of proposed bauxite mining in Andhra Pradesh, India from a quantitative-based environmental impact assessment. Environmental Earth Sciences. 2012;67(6):1587–603.

[24] Giri V. Diversity and conservation status of the Western Ghats amphibians In: Stuart SN, Hoffman M, Chanson JS, Cox NA, Berridge R, Ramani P, et al, editors. Threatened amphibians of the world. Barcelona: Lynx Ediciones, with IUCN-The World Conservation Union, Conservation International and Nature Serve.; 2016.

[25] Aravind N, Gururaja K. Theme paper on the amphibians of the Western Ghats. Report submitted to Western Ghats ecology panel. 2011;20Available from: http://www.westernghatsindia.org/sites/default/files/Amphibians.

[26] Dinesh KP, Radhakrishnan C, Channakeshavamurthy BH, Deepak P, Kulkarni NU. A checklist of Amphibians of India 2017.Available from: http://mhadeiresearchcenter.org/wp-content/uploads/2014/01/2017_April_Checklist-of-Amphibians-of-India.pdf.

[27] Myers N, Mittermeier RA, Mittermeier CG, da Fonseca GA, Kent J. Biodiversity hotspots for conservation priorities. Nature. 2000;403. 10.1038/35002501 10706275

[28] Van Bocxlaer I, Roelants K, Biju S, Nagaraju J, Bossuyt F. Late Cretaceous vicariance in Gondwanan amphibians. Plos One. 2006;1(1):e74. 17183706 10.1371/journal.pone.0000074 PMC1762348

[29] Pyron RA. Biogeographic Analysis Reveals Ancient Continental Vicariance and Recent Oceanic Dispersal in Amphibians. Systematic Biology. 2014;63(5):779–97. doi: 10.1093/sysbio/syu042 24951557 10.1093/sysbio/syu042

[30] Ghalambor CK, Huey RB, Martin PR, Tewksbury JJ, Wang G. Are mountain passes higher in the tropics? Janzen's hypothesis revisited. Integrative and Comparative Biology. 2006;46(1):5–17. doi: 10.1093/icb/icj003 21672718 10.1093/icb/icj003

[31] Ines Van Bocxlaer S, Loader S, Bossuyt F. Toad radiation reveals into-India dispersal as a source of endemism in the Western Ghats-Sri Lanka biodiversity hotspot. BMC evolutionary Biology. 2009;9(1):131. 19519901 10.1186/1471-2148-9-131 PMC2706228

[32] Raj P, Deepak V, Vasudevan K. Monitoring of breeding in Nasikabatrachus sahyadrensis (Anura: Nasikabatrachidae) in the southern Western Ghats, India. Herpetology Notes. 2011;4:11–6.

[33] Hiragond NC, Shanbhag BA, Saidapur SK. Description of the tadpole of a stream breeding frog, Rana curtipes. Journal of Herpetology. 2001;35(1):166.

[34] Humraskar D, Velho N. The need for studies on amphibians in India. Current Science Association, CV Raman Avenue, PO Box 8005, Bangalore 560 080, India; 2007: 1032.

[35] Santos-Barrera G, Urbina-Cardona JN. The role of the matrix-edge dynamics of amphibian conservation in tropical montane fragmented landscapes. Revista Mexicana de Biodiversidad. 2011;82(2):679–87.

[36] Wells KD, Schwartz JJ. The behavioral ecology of anuran communication Hearing and sound communication in amphibians: Springer; 2007.

[37] Duellman WE. Patterns of species diversity in anuran amphibians in the American tropics. Annals of the Missouri Botanical Garden. 1988:79–104.

[38] Lee JC. Geographic variation in size and shape of neotropical frogs: a precipitation gradient analysis: Museum of Natural History, University of Kansas; 1993.

[39] Friend GR, Cellier KM. Wetland herpetofauna of Kakadu National Park, Australia: seasonal richness trends, habitat preferences and the effects of feral ungulates. Journal of Tropical Ecology. 1990;6(02):131–52.

[40] Vonesh JR. The amphibians and reptiles of Kibale Forest, Uganda: herpetofaunal survey and ecological study of the forest floor litter community. MSc Thesis, University of Florida; 1998. Available from: http://etd.fcla.edu/etd/uf/1998/amd0037/masterslast.pdf.

[41] Lynch JDD, Duellman WE. The Eleutherodactylus of the Amazonian slopes of the ecuadorian Andes (Anura: Leptodactylidae). University of Kansas, Museum of Natural History, Miscelaneous Publications, 1980.

[42] Fauth J, Crother B, Slowinski J. Elevational patterns of species richness, evenness, and abundance of the Costa Rican leaf-litter herpetofauna. Biotropica. 1989;21:178–85.

[43] da Silva FR, Almeida-Neto M, do Prado VHM, Haddad CFB, de Cerqueira Rossa-Feres D. Humidity levels drive reproductive modes and phylogenetic diversity of amphibians in the Brazilian Atlantic Forest. Journal of Biogeography. 2012;39(9):1720–32.

[44] Haddad CFB, Prado CPA. Reproductive Modes in Frogs and Their Unexpected Diversity in the Atlantic Forest of Brazil. BioScience. 2005;55(3):207–17.

[45] Iskandar DT, Evans BJ, McGuire JA. A Novel Reproductive Mode in Frogs: A New Species of Fanged Frog with Internal Fertilization and Birth of Tadpoles. Plos One. 2015;9(12):e115884. 10.1371/journal.pone.0115884 PMC4281041 25551466

[46] Krishnamurthy S, Manjunatha RA, Gururaja K. A new species of frog in the genus Nyctibatrachus (Anura: Ranidae) from Western Ghats, India. Curr Sci India. 2001;80(7):887–91.

[47] Seshadri KS, Gururaja KV, Aravind NA. A new species of Raorchestes (Amphibia: Anura: Rhacophoridae) from mid-elevation evergreen forests of the southern Western Ghats, India. Zootaxa. 2012;3410:19–34.

[48] Gaitonde N, Giri V, Kunte K. ‘On the rocks’: reproductive biology of the endemic toad Xanthophryne (Anura: Bufonidae) from the Western Ghats, India. Journal of Natural History. 2016;50(39–40):2557–72.

[49] Molur S, Krutha K, Paingankar MS, Dahanukar N. Asian strain of Batrachochytrium dendrobatidis is widespread in the Western Ghats, India. Diseases of aquatic organisms. 2015;112(3):251–5. doi: 10.3354/dao02804 25590776 10.3354/dao02804

[50] Heard GW, Scroggie MP, Ramsey DSL, Clemann N, Hodgson JA, Thomas CD. Can habitat Management Mitigate Disease Impacts on Threatened Amphibians? Conservation Letters. 2017 doi: 10.1111/conl.12375

[51] Gascon C. Amphibian conservation action plan: proceedings IUCN/SSC Amphibian Conservation Summit 2005: IUCN; 2007.

[52] Amstrup SC, McDonald TL, Manly BF. Handbook of capture-recapture analysis: Princeton University Press; 2010.

[53] Williams SE, Hero J-M. Multiple determinants of Australian tropical frog biodiversity. Biological conservation. 2001;98(1):1–10.

[54] Laurance WF, Carolina Useche D, Shoo LP, Herzog SK, Kessler M, Escobar F, et al Global warming, elevational ranges and the vulnerability of tropical biota. Biological Conservation. 2011;144(1):548–57.

[55] Ernst R, Rödel MO. Community assembly and structure of tropical leaf-litter anurans. Ecotropica. 2006;12:113–29.

[56] Menin M, Waldez F, Lima A. Effects of environmental and spatial factors on the distribution of anuran species with aquatic reproduction in central Amazonia. The Herpetological Journal. 2011;21(4):255–61.

[57] Baillie J, Hilton-Taylor C, Stuart SN. 2004 IUCN red list of threatened species: a global species assessment: Iucn; 2004.

[58] Ramesh V, Gopalakrishna T, Barve S, Melrick DJ. IUCN greatly underestimates threat levels of endemic birds in the Western Ghats. Biological Conservation. 2017;210:205–21.

[59] Hussain QA. Global amphibian declines: a review. International Journal of Biodiversity and Conservation. 2012;4(10):348–57.

[60] Stuart SN, Chanson JS, Cox NA, Young BE, Rodrigues ASL, Fischman DL, et al Status and trends of amphibian declines and extinctions worldwide. Science. 2004;306(5702):1783 doi: 10.1126/science.1103538 15486254 10.1126/science.1103538

[61] Young BE, Stuart SN, Chanson JS, Cox NA, Boucher TM. Disappearing jewels: the status of new world amphibians. Appl Herpetol. 2005;2:429–35.

[62] Feeley KJ, Stroud JT, Perez TM. Most ‘global’ reviews of species’ responses to climate change are not truly global. Diversity and Distributions. 2016 doi: 10.1111/ddi.12433

[63] Deans RA, Chalcraft DR. Matrix context and patch quality jointly determine diversity in a landscape‐scale experiment. Oikos. 2016.

[64] Mortelliti A, Amori G, Boitani L. The role of habitat quality in fragmented landscapes: a conceptual overview and prospectus for future research. Oecologia. 2010;163(2):535–47. doi: 10.1007/s00442-010-1623-3 20414787 10.1007/s00442-010-1623-3

[65] Ficetola GF, De Bernardi F. Amphibians in a human-dominated landscape: the community structure is related to habitat features and isolation. Biological conservation. 2004;119(2):219–30.

[66] Biju S, Bossuyt F. Systematics and phylogeny of Philautus gistel, 1848 (Anura, rhacophoridae) in the Western Ghats of India, with descriptions of 12 new species. Zool J Linn Soc. 2009;155(2):374–444.

[67] Holdridge LR. Life zone ecology. Life zone ecology. 1967(rev. ed.)).

[68] Watve A. Rocky plateaus (special focus on the Western Ghats and Konkan). Report to Western Ghats Ecology Expert Panel: BIOME Conservation Foundation; 2010.

[69] India Go. Indiastat,Meteorogical Data, Rainfall 2017. Available from: http://www.indiastat.com/meteorologicaldata/22/rainfall/238/stats.aspx.

[70] Crump ML, Scott NJ. Visual encounter surveys In: Heyer WR, Donnelly MA, McDiarmid RW, Hayek LC, Foster MS, editors. Measuring and Monitoring Biological Diversity: Standard Methods for Amphibians. Washington: Smithsonian Institution Press; 1994.

[71] Crump ML, Scott NJ. Visual encounter surveys In: Heyer WR, Donnelly MA, McDiarmid RW, Hayek LC, Foster MS, editors. Measuring and Monitoring Biological Diversity: Standard Methods for Amphibians. Washington: Smithsonian Institution Press; 1994.

[72] Daniel J. The Book of Indian Reptiles and Amphibians. Bombay Natural History Society and Oxford University Press Oxford; 2002.

[73] Doan TM. Which methods are most effective for surveying rain forest herpetofauna? Journal of Herpetology. 2003;37(1):72–81.

[74] Vonesh JR, Mitchell JC, Howell K, Crawford AJ. Rapid assessments of amphibian diversity In: Dodd CKJ, editor. Amphibian ecology and conservation: A handbook of techniques. New York, USA: Oxford University Press; 2010.

[75] Babbitt KJ, Veysey JS, Tanner GW. Measuring habitat In: Dodd CKJ, editor. Amphibian Ecology and Conservation: A Handbook of Techniques. New York, USA: Oxford University Press; 2009.

[76] Pounds JA, Fogden MPL, Campbell JH. Biological response to climate change on a tropical mountain. Nature. 1999;398(6728):611–5.

[77] Bhatta G. A field guide to the caecilians of the Western Ghats, India. Journal of biosciences. 1998;23(1):73–85.

[78] Dubois A, Ohler A-M, Biju SD. A new genus and species of Ranidae (Amphibia, Anura) from south-western India. Alytes. 2001;19(2–4):53–79.

[79] Bossuyt F. A new species of Philautus (Anura: Ranidae) from the Western Ghats of India. Journal of Herpetology. 2002;36(4):656–61.

[80] Giri V, Wilkinson M, Gower D. A new species of Gegeneophis Peters (Amphibia: Gymnophiona: Caeciliidae) from the Western Ghats of southern Maharashtra, India, with a key to the species of the genus. Zootaxa. 2003;351:1–10.

[81] Kuramoto M, Joshy SH, Kurabayashi A, Sumida M. The genus Fejervarya (Anura: Ranidae) in central Western Ghats, India, with descriptions of four new cryptic species. Current Herpetology. 2007;26(2):81–105.

[82] Kuramoto M, Joshy SH. Two New Species of the Genus Philautus (Anura: Rhacophoridae) from the Western Ghats, Southwestern India. Current herpetology. 2003;22(2):51–60.

[83] Dinesh K, Radhakrishnan C, Gururaja K, Bhatta G. An annotated checklist of amphibian of India with some insights into the patterns of species discoveries, distribution and endemism. 2009. Available from: https://scholar.google.co.uk/scholar?hl=en&as_sdt=0%2C5&q=Dinesh+K%2C+Radhakrishnan+C%2C+Gururaja+K%2C+Bhatta+G.+An+annotated+checklist+of+amphibian+of+India+with+some+insights+into+the+patterns+of+species+discoveries%2C+distribution+and+endemism&btnG=.

[84] Gower DJ, Mauro DS, Giri V, Bhatta G, Govindappa V, Kotharambath R, et al Molecular systematics of caeciliid caecilians (Amphibia: Gymnophiona) of the Western Ghats, India. Molecular Phylogenetics and Evolution. 2011;59(3):698–707. doi: 10.1016/j.ympev.2011.03.002 21406239 10.1016/j.ympev.2011.03.002

[85] Padhye A, Sayyed A., Jadhav A., Dahanukar N. *Raorchestes ghatei*, a new species of shrub frog (Anura: Rhacophoridae) from the Western Ghats of Maharashtra, India. Journal of Threatened Taxa. 2013;5(15):4913–31.

[86] Frost D, Grant T, Faivovich J, Bain R, Haas A, Haddad CFBdS R, et al The amphibian tree of life. Bull Am Mus Nat Hist. 2006;297:1–370.

[87] Smith H, Chiszar D. Dilemma of name-recognition: Why and when to use new combinations of scientific names. Herpetological Conservation and Biology. 2006;1(1):6–8.

[88] Frost DR. Amphibian Species of the World: an Online Reference New York, USA: American Museum of Natural History; 2015Available from: http://research.amnh.org/herpetology/amphibia/index.html.

[89] Hillis DM. Constraints in naming parts of the Tree of Life. Molecular Phylogenetics and Evolution. 2007;42(2):331–8. doi: 10.1016/j.ympev.2006.08.001 16997582 10.1016/j.ympev.2006.08.001

[90] Clarke KR, Gorley RN. Primer v7: User Manual/Tutorial. Plymouth, UK: Primer-e; 2015.

[91] Anderson MJ, Gorley RN, Clarke KR. Permanova+ for Primer: Guide to software and statistical methods. Plymouth, UK: Primer-e; 2008.

[92] Akaike H. Information theory and an extension of the maximum likelihood principle In: Pertrov BN, Csaki F, editors. 2nd International Symposium on Information Theory. Budapest: Akademiai Kiado; 1973.

[93] Clarke KR, Warwick RM. Change in Marine Communities An approach to statistical analysis and interpretation 2^nd^ edition Plymouth Marine laboratory, Plymouth, UK, Primer-e 1994.

[94] Mahabal A, Sharma RM. Fauna of Maharashtra. Kolkata: Zoological Survey of India; 2012.

[95] IMD IMD. Onset and withdrawal of southwest monsoon 2016: Ministry of Earth Sciences, Government of India; 2016Available from: http://www.imd.gov.in/pages/monsoon_main.php.

[96] Bell EA, Bell BD. Local distribution, habitat, and numbers of the endemic terrestrial frog Leiopelma hamiltoni on Maud Island, New Zealand. New Zealand Journal of Zoology. 1994;21(4):437–42.

[97] Vasudevanl’i K, Kumar A, Chellaml R. Structure and composition of rainforest floor amphibian communities in Kalakad—Mundanthurai Tiger Reserve. Curr Sci India. 2001;80(3).

[98] Gururaja K, Reddy AM, Keshavayya J, Krishnamurthy S. Habitat occupancy and influence of abiotic factors on the occurrence of Nyctibatrachus major (Boulenger) in central Western Ghats, India. Russian Journal of Herpetology. 2013;10(2):87–92.

[99] Krishnamurthy S. Amphibian assemblages in undisturbed and disturbed areas of Kudremukh National Park, central Western Ghats, India. Environmental Conservation. 2003;30(03):274–82.

[100] Vonesh J. Patterns of richness and abundance in a tropical African leaf-litter herpetofauna. Biotropica. 2001;33:502–10.

[101] Naniwadekar R, Vasudevan K. Patterns in diversity of anurans along an elevational gradient in the Western Ghats, South India. Journal of Biogeography. 2007;34(5):842–53.

[102] Cortés-Gómez AM, Castro-Herrera F, Urbina-Cardona JN. Small changes in vegetation structure create great changes in amphibian ensembles in the Colombian Pacific rainforest. Tropical Conservation Science. 2013;6(6):749–69.

[103] Kupfer A, Nabhitabhata J, Himstedt W. From water into soil: trophic ecology of a caecilian amphibian (Genus Ichthyophis). Acta Oecologica. 2005;28(2):95–105.

[104] Lekhak M, Yadav S. Herbaceous vegetation of threatened high altitude lateritic plateau ecosystems of Western Ghats, southwestern Maharashtra, India. Rheedea. 2012;22(1):39–61.

[105] Robin V, Nandini R. Shola habitats on sky islands: status of research on montane forests and grasslands in southern India. Current Science(Bangalore). 2012;103(12):1427–37.

[106] Hanski I. Metapopulation dynamics. Nature. 1998;396(6706):41–9.

[107] Schut AG, Wardell-Johnson GW, Yates CJ, Keppel G, Baran I, Franklin SE, et al Rapid characterisation of vegetation structure to predict refugia and climate change impacts across a global biodiversity hotspot. Plos One. 2014;9(1):e82778 doi: 10.1371/journal.pone.0082778 24416149 10.1371/journal.pone.0082778 PMC3885404

[108] Smith GR, Rettig JE, Mittelbach GG, Valiulis JL, Schaack SR. The effects of fish on assemblages of amphibians in ponds: a field experiment. Freshwater Biology. 1999;41(4):829–37.

[109] Hartel T, Nemes S, Cogălniceanu D, Öllerer K, Schweiger O, Moga C-I, et al The effect of fish and aquatic habitat complexity on amphibians. Hydrobiologia. 2007;583(1):173.

[110] Biju SD. A synopsis to the frog fauna of the Western Ghats, India. Occasional publication of ISCB, 2001.

[111] Gaitonde N, and Giri V. Primitive breeding in an ancient Indian frog genus *Indirana*. Curr Sci India. 2014;107(1):109–12.

[112] Chan LM. Seasonality, microhabitat and cryptic variation in tropical salamander reproductive cycles. Biological Journal of the Linnean Society. 2003;78(4):489–96.

[113] Seshadri KS, Gururaja KV, Bickford DP. Breeding in bamboo: a novel anuran reproductive strategy discovered in Rhacophorid frogs of the Western Ghats, India. Biological Journal of the Linnean Society. 2015;114(1):1–11.

[114] Rittenhouse TA, Semlitsch RD. Distribution of amphibians in terrestrial habitat surrounding wetlands. Wetlands. 2007;27(1):153–61.

[115] Daniels RR. Geographical distribution patterns of amphibians in the Western Ghats, India. Journal of Biogeography. 1992:521–9.

[116] Gower DJ, Wilkinson M. Conservation Biology of Caecilian Amphibians. Conservation Biology. 2005;19(1):45–55.

[117] Oertli B, Joye DA, Castella E, Juge R, Cambin D, Lachavanne J-B. Does size matter? The relationship between pond area and biodiversity. Biological Conservation. 2002;104(1):59–70.

[118] Scheffer M, van Geest GJ, Zimmer K, Jeppesen E, Søndergaard M, Butler MG, et al Small habitat size and isolation can promote species richness: second-order effects on biodiversity in shallow lakes and ponds. Oikos. 2006:227–31.

[119] Jocqué M, Graham T, Brendonck L. Local structuring factors of invertebrate communities in ephemeral freshwater rock pools and the influence of more permanent water bodies in the region. Hydrobiologia. 2007;592(1):271–80.

[120] IPCC. International Panel on Climate Change, Chapter 24: Asia 2014.Available from: http://www.ipcc.ch/pdf/assessment-report/ar5/wg2/WGIIAR5-Chap24.

[121] Frishkoff LO, Hadly EA, Daily GC. Thermal niche predicts tolerance to habitat conversion in tropical amphibians and reptiles. Global Change Biology. 2015;21(11):3901–16. doi: 10.1111/gcb.13016 26148337 10.1111/gcb.13016

[122] Scheffers BR, Edwards DP, Diesmos A, Williams SE, Evans TA. Microhabitats reduce animal's exposure to climate extremes. Global Change Biology. 2014;20(2):495–503. doi: 10.1111/gcb.12439 24132984 10.1111/gcb.12439

