# Supplementary material for: Micro-habitat distribution drives patch quality for sub-tropical rocky plateau amphibians in the northern Western Ghats, India
Source: PLoS One. 2018 Mar 26;13(3):e0194810. doi: 10.1371/journal.pone.0194810 (PMC5868820; doi:10.1371/journal.pone.0194810)
Supplement: S1 Table — (DOCX) [file pone.0194810.s001.docx]

S1 Supplementary information for:

Micro-habitat distribution drives patch quality for sub-tropical rocky plateau amphibians in the northern Western Ghats, India.

Christopher J. Thorpe, Todd R. Lewis, Siddharth Kulkarni, Aparna Watve, Nikhil Gaitonde, David Pryce, Lewis Davies, David T. Bilton and Mairi. E. Knight

**S1 Table 1**. Breakdown of amphibian abundance data by taxa, site, eco-zone, year and day-night time survey.

| **Site** | **Eco-zone** | **Species** | **2013** | **2014 day** | **2014 night** |
| --- | --- | --- | --- | --- | --- |
| Panchgani | High North | *Duttaphrynus melanostictus* | 2 | 1 | 0 |
|  |  | *Fejervarya* (*Zakerana*) cf. *brevipalmata* | 3 | 0 | 0 |
|  |  | *Euphlyctis* cf. *cyanophlyctis* | 0 | 9 | 0 |
|  |  | *Hoplobatrachus tigerinus* | 2 | 3 | 0 |
|  |  | *Raorchestes* cf. g*hatei* | 4 | 0 | 0 |
| Chalkewadi | High North | *Fejervarya* (*Zakerana*) cf. *brevipalmata* | 3 | 4 | 13 |
|  |  | *Duttaphrynus melanostictus* | 1 | 0 | 0 |
| Jagmin | High North | *Euphlyctis* cf. *cyanophlyctis* | 0 | 2 | 0 |
|  |  | *Duttaphrynus melanostictus* | 0 | 4 | 0 |
|  |  | *Raorchestes* cf. *ghatei* | 0 | 2 | 0 |
|  |  | *Indotyphlus maharashtraensis* | 2 | 1 | 0 |
| Mhavashi | High North | *Fejervarya* (*Zakerana*) cf. *caperata* | 0 | 2 | 0 |
|  |  | *Fejervarya* sp. | 2 | 1 | 0 |
| Masai | High Central | *Sphaerotheca dobsonii* | 0 | 1 | 0 |
|  |  | *Hoplobatrachus tigerinus* | 1 | 1 | 0 |
|  |  | *Indotyphlus* cf. *battersbyi* | 3 | 0 | 0 |
| Zender | High Central | *Indirana* cf. *chiravesi* | 3 | 0 | 0 |
|  |  | *Fejervarya* sp. | 0 | 0 | 1 |
| Amboli Low | High South | *Fejervarya* (*Zakerana*) cf. *caperata* | 1 | 2 | 1 |
|  |  | *Duttaphrynus melanostictus* | 0 | 1 | 0 |
|  |  | *Gegeneophis* cf*. ramaswamii* | 6 | 0 | 0 |
|  |  | *Xanthophryne tigerina* | 7 | 7 | 13 |
| Amboli High | High South | *Xanthophryne tigerina* | 30 | 42 | 0 |
|  |  | *Hoplobatrachus tigerinus* | 0 | 1 | 0 |
| Shipole | Low North | *Fejervarya* sp. | 2 | 0 | 0 |
|  |  | *Microhyla ornata* | 0 | 1 | 0 |
|  |  | *Fejervarya sahyadris* | 0 | 5 | 0 |
|  |  | *Hoplobatrachus tigerinus* | 7 | 0 | 0 |
|  |  | *Philautus*sp. | 1 | 0 | 0 |
|  |  | *Pseudophilautus*sp. | 2 | 0 | 0 |
| Ratnagiri | Low Central | *Hoplobatrachus tigerinus* | 5 | 7 | 0 |
|  |  | *Fejervarya sahyadris* | 0 | 3 | 0 |
|  |  | *Uperodon globulosus* | 2 | 0 | 0 |
|  |  | *Sphaerotheca dobsonii* | 1 | 0 | 0 |
| Lanja | Low Central | *Fejervarya* (*Zakerana*) cf. *caperata* | 1 | 0 | 0 |
|  |  | *Hoplobatrachus tigerinus* | 3 | 1 | 1 |
|  |  | *Gegeneophis seshachari* | 0 | 1 | 0 |
|  |  | *Euphlyctis* cf. *cyanophlyctis* | 0 | 1 | 0 |
|  |  | *Polypedates maculatus* | 0 | 1 | 0 |
|  |  | *Sphaerotheca dobsonii* | 0 | 0 | 1 |
|  |  | *Fejervarya* (*Zakerana*) cf. c*epfi* | 9 | 2 | 1 |
|  |  | *Fejervarya sahyadris* | 4 | 1 | 4 |
| Kudopi | Low South | *Gegeneophis seshachari* | 8 | 10 | 0 |
|  |  | *Microhyla ornata* | 0 | 1 | 0 |
|  |  | *Hoplobatrachus tigerinus* | 2 | 2 | 0 |
|  |  | *Fejervarya sahyadris* | 6 | 10 | 0 |
|  |  | *Sphaerotheca dobsonii* | 1 | 0 | 0 |
| Dhamapur | Low South | *Fejervarya* sp. | 10 | 0 | 2 |
|  |  | *Euphlyctis cyanophlyctis* | 0 | 0 | 1 |
|  |  | *Fejervarya sahyadris* | 0 | 8 | 5 |
|  |  | *Fejervarya* (*Zakerana*) cf. c*epfi* | 0 | 0 | 3 |
|  |  | *Sphaerotheca dobsonii* | 0 | 0 | 1 |
|  |  | *Polypedates maculatus* | 0 | 0 | 1 |
|  |  | *Microhyla ornata* | 0 | 0 | 1 |
|  |  | *Hoplobatrachus tigerinus* | 2 | 2 | 0 |
| **Total** |  |  | **136** | **140** | **49** |
